# Editorial: Diagnostic procedures in veterinary microbiology and infectious diseases, volume II

**DOI:** 10.3389/fvets.2023.1203952

**Published:** 2023-05-05

**Authors:** Elisa Rampacci, Valentina Stefanetti, Doreene R. Hyatt, Fabrizio Passamonti

**Affiliations:** ^1^Department of Veterinary Medicine, University of Perugia, Perugia, Italy; ^2^Department of Microbiology, Immunology and Pathology, Colorado State University, Fort Collins, CO, United States

**Keywords:** veterinary microbiology, diagnostic assays, novel diagnostic methods, veterinary infectious diseases, veterinary diagnostic laboratories

Veterinary infectious diseases significantly undermine the health of livestock, domestic animals and wildlife, and, as potential emerging zoonoses, are of great concern for public health. From the recent SARS-COV2 spreading, we have learned the importance of working with a One-Health perspective. As demonstrated by this pandemic, the impact of increasingly frequent epidemic animal diseases demonstrates the importance and urgency of correct diagnostic processes. Various techniques confirming the presence of the microorganism (i.e., cytology, bacteriological culture, fecal examinations, microarrays, antigen assays, immunohistochemical stains, nucleic acid amplification, serological assays, etc.) are available for many veterinary pathogens. However, diagnosis of veterinary infectious diseases is often challenging due to issues concerning test reliability, performance, quality, time required and cost. As a result, no single test is considered the definitive gold standard for most organisms and alternative methods are under investigation.

We were invited to serve as Guest Editors for Volume II of the Research Topic entitled “*Diagnostic procedures in veterinary microbiology and infectious diseases*,” which integrates well with Volume I of this Research Topic (1). This Research Topic was developed to gather suitable articles to promote new advances in veterinary diagnostic microbiology.

In “*Diagnostic procedures in veterinary microbiology and infectious diseases, volume II*” there are five papers (four Original Research Articles and one Data Report Article) covering the following aspects: diagnostic virology and diagnostic procedures for infectious prion and protozoal diseases.

Enteric viral pathogens are highly prevalent in the porcine industry. Among these, porcine deltacoronavirus (PDCoV) and porcine rotavirus type A (PoRVA) are responsible for severe watery diarrhea, vomiting, dehydration and high mortality in piglets, resulting in huge economic losses in pig production. Because of their relevance, researchers investigated novel diagnostic methods based on nucleic acid amplification. Particularly, Shen et al. developed a one-step, closed-tube loop-mediated isothermal amplification (LAMP) procedure for the detection of PDCoV, which is termed cleaved probe-based reverse transcription loop-mediated isothermal amplification (CP-RT-LAMP). A key element of this technique is the use of a cleaved probe that targets the N gene of the PDCoV and activates the enzyme ribonuclease H2, leading to the hydrolytic release of a quencher moiety and thus a detectable amplified signal. This method was shown to be highly sensitive and specific for PDCoV and to shorten the time required for traditional RT-qPCR. Wang et al. focused their efforts on a novel diagnostic procedure for PoRVA. They established a real-time fluorescent reverse transcription recombinase-aided amplification (RT-RAA) assay that directly allows the use of extracted RNA as template for pathogen detection. The method is based on the amplification of conserved sequences of PoRVA VP6 by using 35 bp-primers to increase test specificity. Moreover, being performed at a constant temperature, RT-RAA assay is proposed for rudimentary laboratories where any thermostatic device is available.

Another article in diagnostic virology is based on the paucity of available full-length sequences of Vesicular stomatitis virus (VSV) in public databases. This limited the understanding of the emergence and transmission of the epidemic lineages of VSV serotype Indiana (VSIV), responsible for a 2019 outbreak in the United States, particularly in Colorado. In this context, Bertram et al. generated a collection of 86 near-full-length genomes obtained from VSIV isolates collected from naturally infected horses in 27 counties of Colorado in 2019. Phylogenetic analyses revealed that the 2019 sequences grouped by geographic area in three distinct clusters, representing the epidemic lineages circulating in the United States.

Direct microscopic examination of appropriate samples is the cornerstone for achieving a rapid diagnosis for most parasitic protozoa. However, when organisms are difficult to identify because of low number, small size and altered diagnostic features, or they are hindered by prior treatment, or are not being shed, diagnosis of protozoa infection can be achieved by molecular techniques, which include conventional PCR and real-time PCR on clinical samples or in combination with enrichment cultures. Loy et al. conducted a comparative analysis between the technical performance of qPCR and a reverse transcription real-time PCR (Direct RT-qPCR) approach that allows for the direct detection of *Tritrichomonas foetus*, a relevant obligate parasite of the bovine reproductive tract. Direct RT-qPCR was found to be equivalent or superior to qPCR, with 100% agreement on 10 parasites/extraction samples and a limit of detection of 1 parasite/extraction. Additionally, *T. foetus* RNA stability was evaluated in different media and conditions (time and temperature stability). Phosphate-buffered saline was not significantly different from *T. foetus* transport media. Samples are recommended to be held for up to 5 days, and at −20°C for up to 14 days while maintaining detection limits that are representative of naturally infected bulls.

Finally, our Research Topic also includes an original research article on molecular diagnosis of Chronic Wasting Disease (CWD), which is an infectious prion disease affecting cervids. Tewari et al. evaluated the use of real-time quaking-induced conversion (RT-QuIC) to detect CWD in fecal and recto-anal mucosal-associated lymphoid tissue (RAMALT) samples from naturally infected farmed white-tailed deer (*Odocoileus virginianus*) herds at post-mortem. The RT-QuIC assay measures fluorescence from Thioflavin T binding to amyloid fibrils of newly converted recombinant abnormal prion protein. RT-QuIC detected the presence of CWD prions in RAMALT with 100% specificity and 85.7% sensitivity and, in feces, with 100% specificity and 60% sensitivity. These findings lay the foundation for the diagnosis of the CWD status of live animals. The authors also demonstrated, by genotyping, that the most frequent prion protein gene variant in the naturally infected farmed white-tailed deer examined was genotype 96GG, followed by genotype 96GS.

In conclusion, manuscripts that have been published in this volume have described different diagnostic procedures proposed to be better methods to detect the etiological agents of clinically relevant viral, protozoal and prion diseases in animals.

Overall, the insufficient funding in the veterinary sector and the long time required to develop novel diagnostic methods make the advancement of knowledge complicated. Therefore, increasing research efforts is crucial to provide accurate diagnostic strategies for controlling and eradicating animal diseases and zoonoses.

## Author contributions

All authors listed have made a substantial, direct, and intellectual contribution to the work and approved it for publication.
